# An integrated experimental and modeling approach to propose biotinylated PLGA microparticles as versatile targeting vehicles for drug delivery

**DOI:** 10.1186/2194-0517-2-3

**Published:** 2013-02-13

**Authors:** Olivia Donaldson, Zuyi Jacky Huang, Noelle Comolli

**Affiliations:** Villanova University, 800 East Lancaster Avenue, Villanova, PA 19085 USA

## Abstract

Polymeric microparticles with covalently attached biotin are proposed as versatile targeting vehicles for drug delivery. The proposed microparticles made of 85/15 poly (lactic-*co*-glycolic acid) (PLGA) will have biotin available on the outside of the particle for the further attachment with an avidin group. Taking advantage of biotin’s high affinity for avidin, and avidin’s well-known chemistry, the particle has the potential to be easily coated with a variety of targeting moieties. This paper focuses on the design and resulting effect of adding biotin to PLGA microparticles using an integrated experimental and modeling approach. A fluorescent-tagged avidin (488-streptavidin) was used to confirm the presence and bioavailability of biotin on the outside of the particles. For the purpose of this study, bovine serum albumin (BSA) was used as a model therapeutic drug. Microparticles were created using two different types of polyvinyl alcohol 88 and 98 mol% hydrolyzed, which were then analyzed for their size, morphology, and encapsulation capacity of BSA. Release studies performed *in vitro* confirmed the slow release of the BSA over a 28-day period. Based on these release profiles, a release kinetics model was used to further quantify the effect of biotinylation of PLGA microparticles on their release characteristics by quantitatively extracting the effective drug diffusivity and drug desorption rate from the release profiles. It was found that the biotinylation of the PLGA microparticles slowed down both the drug desorption and drug diffusion process, which confirmed that biotinylated PLGA microparticles can be used for controlled drug release. The presented technology, as well as the proposed integrated experimental and modeling approach, forms a solid foundation for future studies using a cell-specific ligand that can be attached to avidin and incorporated onto the microparticles for targeted delivery.

**Electronic supplementary material:**

The online version of this article (doi:10.1186/2194-0517-2-3) contains supplementary material, which is available to authorized users.

## Background

Polymeric microparticles have been widely researched for their ability to serve as controlled drug delivery vehicles Brannon-Peppas (1995; Cleland 1997; Shive and Anderson 1997). The main goals of these vehicles are to provide improved drug disposition Putney (1998), protection from metabolic degradation Dziubla et al. (2005), and increased circulation time Putney (1998). The new goal in these designs, however, is to take them a step further and incorporate a method for targeting specific cells Brannon-Peppas and Blanchette (2004; Fung and Saltzman 1997). It is well known that the side effects of drugs stem from drug interactions in non-targeted cells, such as severe anemia experienced by cancer patients Balkwill (2004; Pegram et al. 1997) and those on antiviral medications (such as HIV and HCV treatments) Sayce et al. (2010). A method to target these cells directly would not only increase the potency of the drugs, but also drastically improve the quality of life for the patients on these treatments.

While biodegradable polymeric microparticles have been investigated for the controlled release of anticancer therapeutics Fung and Saltzman (1997; Datta et al. 2006; Folger et al. 2006), and to a limited extent, for antiviral drugs Datta et al. (2006), the challenge of effective targeting still remains. There are few markers that are specific only to tumor cells, but rather most are simply upregulated and therefore more prevalent Balkwill (2004; Pegram et al. 1997). The challenge of identifying a sole marker to a tumor cell or virus is one that biologist and biochemist are still researching. With an evolving field of possible targets and ligands, the challenge for the engineers then is to create a robust mechanism for incorporation of these new ligands to a polymeric delivery vehicle.

Polymers most commonly used for microparticle drug release include poly(lactic acid) (PLA), poly(glycolic acid) (PGA) and their copolymer poly(lactic-*co*-glycolic acid) (PLGA) Anderson and Shive (1997; Cao and Shoichet 1998; Panyam et al. 2003). In order to obtain the desired release of the drug for the specific delivery, the amount of glycolic acid can be increased to increase the degradation rate, and therefore speed the release time. The PLGA used was an 85/15 mixture of lactic to glycolic acid. The biodegradation of PLGA occurs through a homogenous hydrolytic chain cleavage mechanism, in which both the surface and the bulk polymer degrade at similar rates Anderson and Shive (1997). The breakdown of PLGA is purely through hydrolysis and does not need the assistance of an enzyme Muthu (2009).

In order to target specific cell lines, a robust targeting strategy is proposed. Taking advantage of the affinity of biotin with avidin, a strong non-covalent bond can easily be created by adding biotin to the surface of the PLGA. Biotin strongly binds to avidin and streptavidin via a combination of van der Waals and hydrophobic interactions. In perfect conditions, a single molecule of avidin would bind to four molecules of biotin. This high affinity makes it possible to have site-specific microparticles that have predetermined antibodies attached to the avidin Datta et al. (2006; Moro et al. 1997). This platform would allow for various avidin-antibody complexes to be connected to the biotin microparticle in order to make a multi-faceted drug delivery system. Since this takes advantage of the same mechanism many biochemical assays use, the chemistry of avidin attachment to an antibody, or antibody fragment, is already well known Moro et al. (1997; Diamandis and Christopoulos 1991; Kocbek et al. 2007). This platform allows both targeted delivery (via the PEG-biotin-avidin), as well as controlled release, and biochemical protection of the drug during the delivery via the PLGA.

Previous research has shown the potential in using polymeric microparticles with a similar linkage but using the reverse order (avidin linked to the polymer) Park et al. (2011). The proposed method is used as a simpler method for attaching the conjugate covalently to the polymer while controlling the length of the ‘tethering’ arm spacing the conjugate and the polymer. The proposed tethering arm in this case will be a short chain polyethylene glycol (PEG) that can be increased or decreased in length as needed. The attachment of biotin-PEG to a nanoparticle of PLGA was previously done using a more complex chemistry by Weiss et al., for the proposed use of rapid fluorescent tagging of the nanoparticles Weiss et al. (2007). Although these particles were evaluated for their ability to attach to biotin, the release of drug was not investigated.

In order to better understand the effect that biotin has on the polymeric microparticle, along with the usual *in vitro* characterization (drug encapsulation, morphology, release rates), release kinetics will further be modeled from experimental data to extract important quantitative information that is essential for the comparative study of the proposed PLGA microparticles. Specifically, drug release from polymeric microparticles undergoes two main phases: (1) the induction (or burst) phase in which an initial burst of protein release is observed due to the desorption of proteins from the surface of mesopores within microparticles and the outer surface of microparticles; (2) the diffusion phase in which the macromolecular drug contained in the occlusions of microparticles diffuse through the pores that are formed during the hydration, degradation and erosion of microparticles. Accordingly, the drug desorption rate determines the dynamics of the initial drug burst, while the diffusion rate determines the subsequent drug release. In this work, a theoretical model of macromolecular drug release presented by Batycky et al. (1997), as shown in Equation (1), is used to quantify drug desorption rate and effective drug diffusivity from drug release profiles. Thus, the effect of polyvinyl alcohol (PVA) surfactants as well as the attachment of biotin to the polymeric microparticles on the drug release process can be quantified.

The mass fraction of released drug, *f*_release_, is determined by the following equation:

1 $$\begin{array}{l} f_{\text{release}}=\varphi_{d}^{\text{burst}}\left(1-e^{-k_{d}t}\right)\\ \;+\left(1-\varphi_{d}^{\text{burst}}\right)\left(1-\frac{6}{\pi^{2}}\sum_{j=1}^{\infty }\frac{-e^{-j^{2}\pi^{2}{\overset{―}{D}}_{d}^{*}\left(t-t_{d}\right)/r_{0}^{2}}}{j^{2}}\right) \end{array}$$

where $\varphi_{d}^{\text{burst}}$ is the mass fraction of drug involved in the burst phase, *k*_d_ is the drug desorption rate constant, $\bar{D_{d}^{*}}$ is the effective drug diffusivity, *t*_d_ is the drug induction time that allows for the coalescence of micropores and the passage of the macromolecular drug out from the occlusions through the coalescing micropores in microparticles, and *r*_0_ is the initial microparticle radius. The first term of Equation 1 represents the burst phase during which the proteins from the surface of mesopores within microparticles and the outer surface of microparticles are released and during which the micropores within microparticles coalesce for the further release of the encapsulated drug. Following the burst phase is the diffusion phase that is described by the second term of Equation 1 in a Fickian-release manner. The time evolution of released mass, *f*_release_ predicted from Equation 1, will be compared to the experimental released profile. The values of the parameters that are important for characterizing drug released process, including $\varphi_{d}^{\text{burst}}$, *k*_d_, and *t*_d_, are then determined by fitting the model given in Equation 1 to the experimental data. Therefore, Equation 1 is used as a soft-sensor in this work for quantitatively monitoring the drug desorption rate and effective drug diffusivity that cannot be directly determined from the release profiles by eye inspection. These parameters can be used as quantitative criteria for the selection of PLGA microparticles for drug delivery. The primary goal of this paper is to evaluate the effect of the biotinylation of the PLGA microparticles on their morphology and release characteristics.

## Materials and methods

### Materials

PLGA was purchased from SurModics, located in Birmingham, AL, USA. The EZ-Link®TFPA-PEG_3_-Biotin, 488-streptavidin, potassium nitrate, and micro bicinchoninic acid (BCA) protein assay kit were all obtained from Thermo Fisher Scientific (Waltham, MA, USA). A biotin quantification kit was bought from Pierce Biotechnology (Rockford, IL, USA). The ethyl acetate, dichloromethane (DCM), and dimethyl sulfoxide (DMSO) used in the preparation of the microparticle, as well as bovine serum albumin (BSA) and phosphate buffered solution (PBS) were purchased from Sigma-Aldrich (St. Louis, MO, USA). The sodium azide was purchased from Acros Organics (Geel, Belgium). The PVA was brought from Polysciences, Inc. (Warrington, PA, USA).

### Biotinylation of PLGA

The PLGA and DMSO were combined in a 10:1 ratio and vortexed until the PLGA dissolved. EZ-Link TFPA-PEG3-Biotin was attached to PLGA in a 20-fold molar excess of biotin (10 mg/mL in DMSO). The amount of biotin was determined using the following equation:

2 $$V_{\text{biotin}}=1,000\times \frac{m_{\text{PLGA}}MW_{\text{biotin}}}{MW_{\text{PLGA}}M_{\text{biotin}}C_{\text{biotin}}}$$

where *m*_PLGA_ is the mass of PLGA; *MW*_biotin_ and *MW*_PLGA_ are the molecular weight of biotin and PLGA, respectively; *M*_biotin_ is the mole of excess of biotin; *C*_biotin_ is the concentration of biotin.

The mixture was then photoactivated using UV light for 30 min. After quenching the reaction with approximately 15 mL of deionized (DI) water, the solution was then centrifuged using the Sorvall Legend RT Plus Centrifuge (Thermo Scientific) at 14,000 rpm for approximately 7 h at room temperature. The samples were stored at 4°C until use.

### Quantification of biotin

A biotin quantification kit was used to compare the absorbance of a sample to a positive control, biotinylated horseradish peroxidase (HRP). To begin the analysis, a PLGA-biotin pellet was dissolved in ethyl acetate. 4^′^-hydrocyazobenzene-2-carboxylic acid (HABA)-avidin was then added to both the control and sample. The plate was shaken for approximately 60 s, and the displaced HABA was measured using a BioTek ELx800 UV/Vis microplate reader at a wavelength of 490 nm. The ratio of biotin to PLGA was determined using the recorded absorbance values. All results are presented as the average of triplicate samples with the standard deviation.

### PLGA microparticle synthesis

The water-in-oil-in-water method is a common emulsion technique that was performed at room temperature. Briefly, 150 μL of phosphate buffered saline (pH 7.4) with varying amounts of dissolved protein was added to 2 mL of the oil phase (10 mg/mL PLGA in ethyl acetate), and the emulsion was created by adding energy to the solution by homogenizing for 60 s. The primary emulsion was stabilized with the addition of bovine serum albumin (1 mg/mL) to the internal aqueous phase. The primary emulsion was then quickly added to 300 mL of an external aqueous phase (5 wt.% PVA). The emulsion was stabilized through stirring at 500 rpm and the presence of the PVA (either 88 or 98 mol% hydrolyzed). The microparticles hardened while stirring overnight and the ethyl alcohol was evaporated. The microparticles were collected via centrifugation at 14,000 rpm for 90 min. Afterwards, the supernatant was removed and the microparticles were resuspended in DI water. The microparticles were washed two more times and centrifuged at 14,000 rpm at respectively 90 and 30 min. The microparticles were allowed to dry and either used immediately or kept at 4°C until use.

### Fluorescent imaging

PLGA-biotin and non-biotinylated PLGA particles were analyzed under fluorescent imaging. Approximately 5 mg of particles were suspended in 1 mL of DI water in an amber microcentrifuge tube. A 5 μL of 488-streptavidin was added to the solution and it was stored in a dark location for at least 90 min. The tube was then centrifuged at 13,300 rpm for 15 min at room temperature. Microparticles were then washed, removing the supernatant, and the pellet was resuspended in a small amount of DI water. The sample was centrifuged at 13,300 rpm for 15 min and the supernatant was removed. The sample was then placed on a glass slide with a cover slip and viewed on a Leica DM 2000 microscope (Leica Microsystems, USA). Images were captured and viewed using a Q imaging Retiga-SRV camera and QCapture Pro 6.0 software (Q Imaging, Surrey, British Columbia, Canada).

### Encapsulation efficiency

Protein encapsulation was evaluated by dissolving a known weight of particles (5 mg) in 2 mL DCM. The dissolved particles were mixed with 3 mL of DI water and the solvent-water mixture was shaken overnight at 200 rpm. This provided sufficient time for the protein to be extracted into the water phase. A sample was taken from the water phase and the concentration was found using a BCA protein assay (used per manufacturer’s instructions). The BCA assay is a colorimetric assay based on bicinchoninic acid and measures the total protein content in a sample. Negative controls of the particle made with no protein present at all were also performed to ensure that the presence of the degraded lactic acid did not affect the concentration readings.

### *In vitro* release of model drugs

Protein release from the microparticles was evaluated *in vitro* using a known mass of dried microparticles in 30 mL of PBS (with 0.01% NaN3 to prevent bacterial growth). All studies were set so sink conditions would be maintained, specifically, that at no point would the maximum released concentration of protein be greater than 10% the saturation limit for that protein in PBS. Samples of the release medium were removed at designated times using a sample probe with an inline 0.45-μm filter to prevent removal of the microparticles during sampling. Equal volumes of fresh PBS were back-flushed through the filter to ensure a constant volume throughout the study as well as to ensure that any microparticles trapped in the sample probe would be flushed back into the sample container. Samples were kept at -20°C until analysis. Concentrations were found using the BCA protein assay at 490 nm, per manufacturer’s instructions.

### Particle morphology

Using completely dried microparticles, the size and surface morphology of the particles were observed using the scanning electron microscope. A fraction of the microparticle was taken and placed on a small metal stage, fitted with double-sided carbon tape. The sample was then coated. The sample was placed in a Hitachi S-570 scanning electron microscope (Hitachi America Ltd., Brisbane, CA, USA) for observation under vacuum. The size and distribution of the particles was determined. Using dissolved microparticles, the size of the particle was observed using the Hitachi 7600 transmission electron microscope. Six microliters of the sample was placed on a carbon graph and allowed to dry. The dried sample was sputter coated with a conductive metal and placed in the microscope for examination.

### Particle size

Using completely dried microparticles, the size and polydispersity of the particles were observed using the particle size analyzer. A portion of the microparticle was suspended in 4.5 mL of 10 mM of KNO_3_. The suspension was approximately 10 mg to 4 mL. The sample was then placed in a Brookhaven 90 plus particle size analyzer (Brookhaven Instruments Corporation, Holtsville, NY, USA) for examination. The KNO_3_ was used instead of PBS since the salt solution was necessary to allow for the laser scattering that is needed.

### Quantifying drug desorption rate constant and effective drug diffusivity from drug release profiles

While the BSA release percentage can be determined from the release profile directly, the release kinetics parameters such as the effective diffusion rate and the drug desorption cannot be directly determined by eye inspection from experimental data. Thus, release kinetics parameters such as drug desorption rate (*k*_d_) and effective drug diffusivity $\overset{―}{D_{d}^{*}}$ in Equation 1 were estimated by fitting *f*_release_ predicted by Equation 1 to experimental drug release profiles (‘*In vitro* release of model drugs’ section) via the following procedure:

1. $\varphi_{d}^{\text{burst}}$ and *t*_d_ , which correspond to the mass and time for drug release in the burst phase respectively, were determined from the inflection point of drug release profiles, as the inflection point indicates the switch of drug release from the burst phase to the diffusion phase.
2. *k*_d_ and $\bar{D_{d}^{*}}$ were determined via a nonlinear least squares approach, which can be represented by Equation 3. This computation was performed by minimizing an objective function consisting of the sum of the squares over *N* measurements of the differences between the experimental data *f*_release_ and the model-predicted output *f*_release_. MATLAB (Mathworks Inc., Natick, MA, USA) routine fmincon was used for solving this parameter estimation problem:
  3 $$\begin{array}{l} \underset{k_{d},{\bar{D}}_{d}^{*}}{\text{Min}}\sum_{i=1}^{N}{\left[{\hat{f}}_{\text{release}}\left(i\right)-f_{\text{release}}\left(i\right)\right]}^{2}\\ \text{subject}\;\mathrm{to}\\ f_{\text{release}}=\varphi_{d}^{\text{burst}}(1-e^{-k_{d}t})+(1-\varphi_{d}^{\text{burst}})\\ \;\left(1-\frac{6}{\pi^{2}}\sum_{j=1}^{\infty }\frac{-e^{-j^{2}\pi^{2}{\bar{D}}_{d}^{*}\left(t-t_{d}\right)/r_{0}^{2}}}{j^{2}}\right)\text{.} \end{array}$$

## Results and discussion

**Figure 1:**
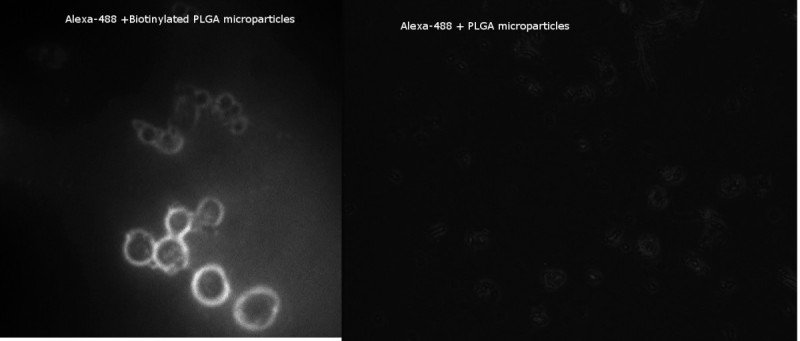
**Fluorescent imaging confirms presence of biotin on the outside of microparticles.** The presence and bioavailability of biotin on the surface of the PLGA microparticles was confirmed by incubating biotinylated microparticles with streptavidin-488. The particles were washed several times prior to visualization under the fluorescent microscope. Left image, Alexa 488 + biotin-PLGA microparticles. The presence of the 488 fluorophore (white) indicates the biotin was present and capable of attaching the steptavidin. Right image, Alexa 488 + PLGA microparticles.

The first step in microparticle production was to synthesize biotinylated PLGA. To do this, TFPA-PEG_3_-Biotin was attached using a UV-initiated reaction. In order to confirm the attachment of the biotin on the polymer, a biotin quantification kit was used. The weight of biotin in relation to polymer was calculated and found to be approximately 0.87 ± 0.37. Once the polymer was confirmed to have biotin, microparticles were made using a water-in-oil-in-water (W/O/W) method. To ensure that the biotin was available to avidin on the outside of the polymer microparticle, a fluorescent assay was performed. Microparticles were combined with streptavidin that was tagged with a green fluorophore (Alexa 488; Life Technologies Corporation, Carlsbad, CA, USA). After incubation, the microparticles were collected, washed, and immediately viewed using a Leica DM 2000 microscope. Presence of green fluorophore (white in Figure 1, Alexa 488 + biotin-PLGA microparticles) around the microparticle indicates that the biotin not only attached to the perimeter, but also was still biologically available for the streptavidin post-microparticle processing. A negative control (Figure 1, Alexa 488 + PLGA microparticles), confirms that the streptavidin is not merely sticking to the polymer surface, but rather to the biotin available on the surface.

Once the microparticle synthesis viability was confirmed, the size and polydispersity were confirmed using a Brookhaven 90 plus particle size analyzer (via dynamic light scattering). Microparticles were made with and without biotin, as well as with and without the model drug (BSA). Microparticles were also made using two different types of PVA (the surfactant for the secondary emulsion step, 88 and 98 mol% hydrolyzed) in an attempt to optimize the microparticle synthesis. Analysis of the samples (Table 1) found that the addition of biotin to the PLGA causes an increase in particle size for both 88 and 98 mol% hydrolyzed PVA. This trend is expected since the presence of the biotin makes the polymer larger as well as more hydrophobic. The biontinylated PLGA particle containing no BSA created a smaller distribution in the molecular masses of the samples. This could be a result of the biotin itself helping to stabilize the emulsion. Interestingly, the addition of BSA without biotin present increased the particle size; however, the addition of BSA into the biotinylated PLGA particles actually showed a decrease in size. This decrease in size was unexpected but may indicate a specific reaction of the BSA and biotin during the W/O/W emulsion and may not be critical when using the actual cancer therapeutic drug. The particles were created using both 88 and 98 mol% PVA, and it was found that particles generated from the 98 mol% PVA are larger than the particles made from the 88 mol% PVA. The 98 mol% PVA is roughly ten times larger than the 88 mol% PVA particles. The polydispersity of the 98 mol% PVA microparticles was larger than the 88 mol% PVA microparticles as well, indicating that the increase in hydrophilicity in the 98 mol% PVA did not provide an increased stabilizing effect on the emulsion as compared to the 88 mol% PVA.

**Table 1:** **Average size and polydispersity of different microparticle formulations**

Type (mol% PVA)	Average size (μm)	Average (PDI) polydispersity index
PLG no BSA (88)	7.81 ± 13.52	0.48 ± 0.55
PLGA BSA (88)	8.06 ± 23.12	0.36 ± 0.037
Biotin BSA (88)	1.83 ± 4.23	0.39 ± 0.18
Biotin no BSA (88)	21.24 ± 15.02	0.021 ± 0.015
PLGA No BSA (98)	21.99 ± 14.91	0.67 ± 0.39
PLGA BSA (98)	30.72 ± 49.99	0.50 ± 0.36
Biotin BSA (98)	16.47 ± 11.85	0.80 ± 0.36
Biotin No BSA (98)	37.97 ± 97.51	0.39 ± 0.52

Microparticles were synthesized using either 88 or 98 mol% PVA as a surfactant as well as with and without the biotin attached to the PLGA. The resulting microparticle size and polydispersity were determined via dynamic light scattering. Results are presented as the average of *n* = 4 samples ± standard deviation.

**Figure 2:**
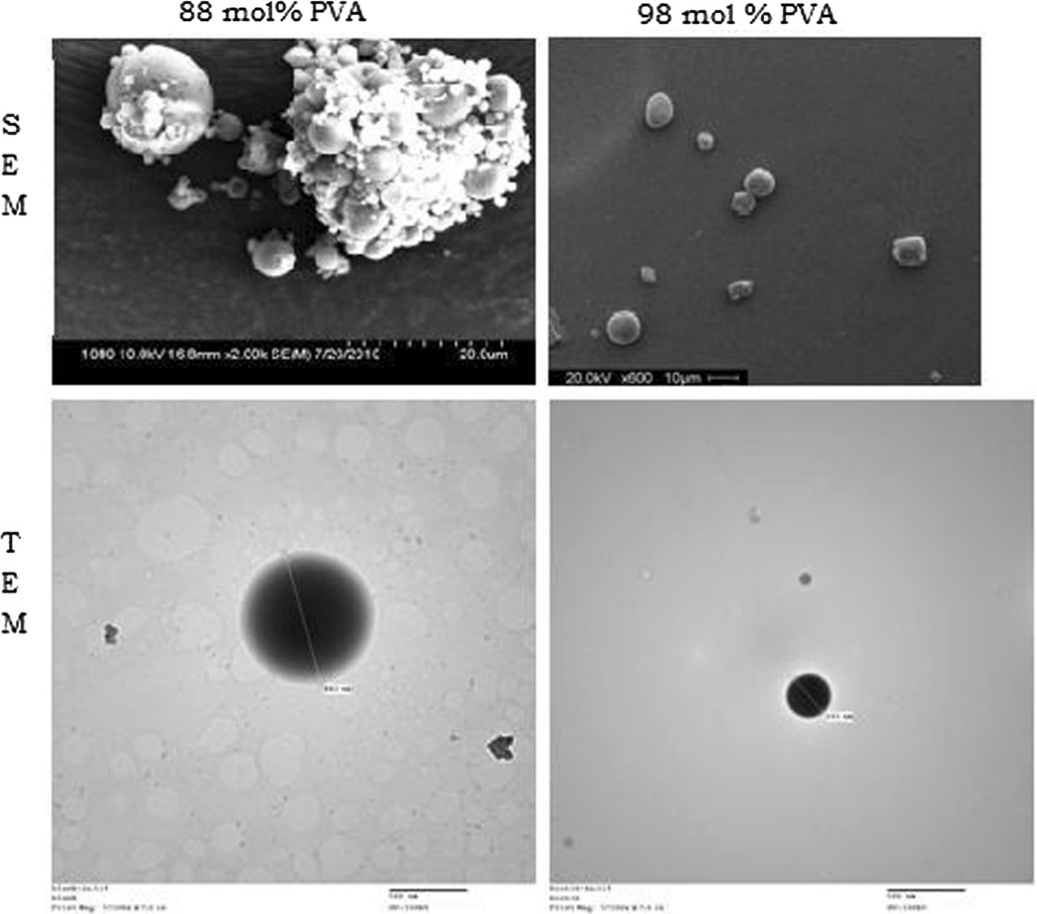
**Microparticle size and morphology.** Biotinylated microparticles were synthesized using either 88 or 98 mol% PVA as a surfactant. The resulting microparticles were imaged using a SEM (top images) as well as a TEM (bottom images). Images confirmed the spherical morphology and size ranges determined via dynamic light scattering.

In order to evaluate the morphology of the microparticles, both a Hitachi S-4800 scanning electron microscope (SEM) and a Hitachi 7600 transmission electron microscope (TEM) were used to view samples of the biotinylated and plain PLGA microparticles. SEM and TEM images of both 88 and 98 mol% PVA methods are shown in Figure 2. The TEM and SEM images confirm that the addition of the biotin to the PLGA does not change the spherical morphology of the microparticles. The microparticles with and without biotin do not have any morphological differences visible. The particles also have no visible morphological changes (with the exception of size) when changing from 88 to 98 mol% PVA during processing. The ability of the microparticle to keep its spherical shape is an indication that the microparticle morphology is not changed by the addition of the biotin. The agglomeration seen in some of the SEM is believed to be a result of the drying process for imaging and is not expected when the microparticles are in solution.

Once the particle size and morphology was characterized, the mass percentage of BSA encapsulated as well as the encapsulation efficiency of the model drug was determined (Table 2). It was found that for particles made from 88 mol% PVA, the PLGA particles had a slightly higher average encapsulation than the biotinylated particles. On the other hand, when the PVA was changed to 98 mol%, the average encapsulation percentage for the biotinylated particle was higher than the PLGA particle. The 88 mol% PVA, however, had similar encapsulation of BSA between the plain PLGA and biotinylated PLGA microparticles. Although the 98 mol% hydrolyzed PVA may be a better surfactant, there is no clear trend apparent for the effect of biotin on the encapsulation of a drug; further optimization should be done using the actual therapeutic. It was determined that the 98 mol% PVA more effectively stabilized the microparticle during hardening, allowing for the higher encapsulation (shown in Table 2) of BSA. If the particle can be successfully stabilized during the hardening step, it should not swell and allow water in, or drug out. If higher encapsulation corresponds to the larger size, since more internal aqueous phase is retained, the microparticles should be larger. The movement of drug and water in and out during this phase is a common problem during hardening, since the osmotic pressure will readily drive molecules through the oil phase (polymer in solvent) until it fully hardens. This flux creates the largest challenge in designing microparticle with a high drug loading.

**Table 2:** **BSA loading for different microparticle formulations**

Type (mol% PVA)	Average mass encapsulation percentage	Average encapsulation efficiency
PLG (88)	4.12	3.21
Biotin (88)	3.30	1.78
PLGA (98)	3.02	4.35
Biotin (98)	12.26	15.57

Microparticles were synthesized using either 88 or 98 mol% PVA as a surfactant as well as with and without the biotin attached to the PLGA. The resulting microparticle’s loading of BSA was quantified dissolving the microparticle and extracting the BSA which was quantified using a micro BCA assay. The BSA loading is presented as both the percentage of the particle mass that is BSA as well as the percentage of the initial BSA that was actually loaded into the microparticle.

**Figure 3:**
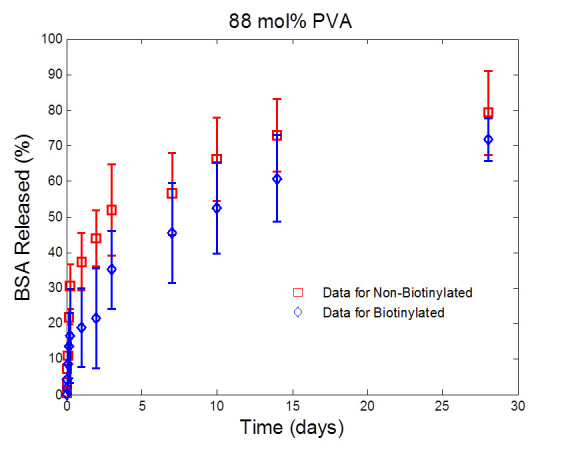
**BSA release from microparticles synthesized with 88 mol% PVA.** BSA release from microparticles synthesized using 88 mol% PVA (with and without biotin) *in vitro* over a 28-day period. Both the biotinylated and non-biotinylated microparticles controlled the release of the BSA with a minimal initial burst. Samples were quantified using the micro BCA and are presented as the average of *n* = 4. Error bars indicate the standard deviation. No difference was found between the two sets of microparticles release profiles.

**Figure 4:**
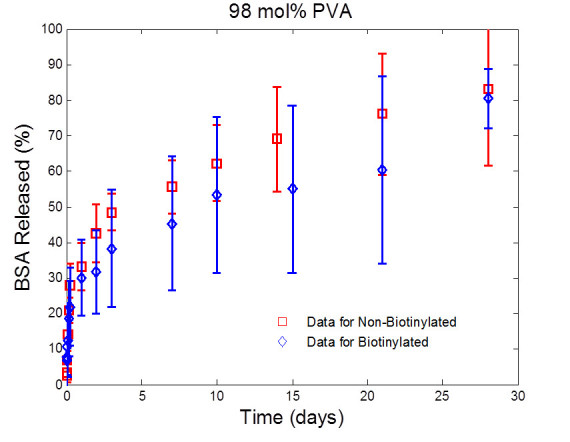
**BSA release from microparticles synthesized with 98 mol% PVA.** BSA release from microparticles synthesized using 98 mol% PVA (with and without biotin) *in vitro* over a 28-day period. Both the biotinylated and non-biotinylated microparticles controlled the release of the BSA with a minimal initial burst, with no real difference in their release profiles. Samples were quantified using the micro BCA and are presented as the average of *n* = 4. Error bars indicate the standard deviation.

Once the amount of drug encapsulated in each type of microparticle was determined, the rate of release of the model drug could be evaluated. *In vitro* release studies were performed under sink condition in PBS at 37°C for all four types of microparticles. Using a micro BCA assay, the mass of BSA released was determined. The cumulative percentage of BSA released over time was calculated and is shown in Figures 3 and 4. It was determined that the release of BSA from the biotinylated and PLGA particle, made from 88 mol% PVA, followed the same trend (Figure 3) over a 28-day period. This indicates that the presence of biotin on the surface of the microparticle does not alter the release characteristics of the microparticles. The same result was found for microparticles made using 98 mol% PVA (Figure 4). For the 28-day period, approximately 80% of the model drug is released for both biotinylated and non-biotinylated microparticles. Comparing Figures 3 and 4, there is no apparent effect of the change in surfactant on release, as expected. The change in the surfactant should mainly change the microparticles stability during formation, leading to potential changes in morphology and encapsulation capacity.

**Figure 5:**
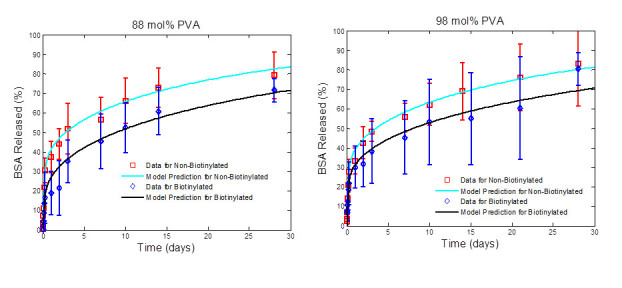
**Comparison of experimental data to BSA release profiles.** Comparison of experimental data to BSA release profiles, predicted by the model shown in Equation 1 with estimated parameters $\bar{D_{d}^{*}}$, *k*_d_, *φ*_*d*_^*burst*^, and *t*_d_. The RMSD values for microparticles PLGA-BSA (88 mol% PVA), biotin-BSA (88 mol% PVA), PLGA-BSA (98 mol% PVA), and biotin-BSA (98 mol% PVA) are 2.68%, 4.05%, 2.37%, and 4.55%, respectively. Error bars indicate the standard deviation.

In order to further quantify the effect of the biotinylation of the PLGA microparticles on the drug release characteristics, the effective drug diffusivity and drug desorption rate that directly characterize the drug release process are further determined from the release profiles. Specifically, the model given by Equation 1 is fitted to the release profiles presented in Figures 3 and 4 via the approach shown in ‘Quantifying drug desorption rate constant and effective drug diffusivity from drug release profiles’ section. The values of the parameters $\overset{―}{D_{d}^{*}}$, *k*_d_, $\varphi_{d}^{\text{burst}}$ and *t*_d_ in Equation 1 are estimated. The fitting result is presented in Figure 5, which shows that the drug release profiles predicted by the estimated model fit the experimental data well for all types of microparticles under investigation. In particular, the predicted drug release profiles pass through most error bars shown in the data. The root-mean-square deviation of prediction (RMSD) for microparticles PLGA-BSA (88 mol% PVA), biotin-BSA (88 mol% PVA), PLGA-BSA (98 mol% PVA), and biotin-BSA (98 mol% PVA) is calculated as 2.68%, 4.05%, 2.37%, and 4.55%, respectively. As shown in Figure 5, the model predicts that more BSA is released by the plain PLGA microparticles than the biotinylated PLGA microparticles, and that PLGA microparticles made of a lower percent of PVA release slightly more BSA. This is in a good agreement with the trends shown in the release profiles. A conclusion drawn from these observations is that the deviation of the model prediction from the experimental data is within a reasonably small scale, and that values of parameters $\bar{D_{d}^{*}}$, *k*_d_, $\varphi_{d}^{\text{burst}}$, and *t*_d_ estimated from release profiles properly characterize the drug release dynamics of all microparticles under investigation.

Table 3 shows the corresponding estimated values of parameters $\bar{D_{d}^{*}}$, *k*_d_, $\varphi_{d}^{\text{burst}}$, and *t*_d_. It can be seen from Table 3 that $\bar{D_{d}^{*}}$ decreases by a factor of 0.65 when microparticles made of 88 mol% PVA were biotinylated. A similar decreasing ratio (i.e., 0.61) is observed in the value of $\bar{D_{d}^{*}}$ for biotinylated microparticles that are made of 98 mol% PVA. This means that the attachment of biotin to microparticles slightly slows down the drug diffusion and thus the drug release process. This is expected since the presence of the biotin on the outer surface acts as another layer of diffusion barrier. The value of $\bar{D_{d}^{*}}$ decreases by a factor of approximately 0.90 when 98 mol% PVA instead of 88 mol% PVA is used to make microparticles. This implies that increasing the mole percentage of PVA can slightly slow down drug release but to a limited degree. The decrease in drug release at higher mole percentage of PVA may be due to its tendency to remain on the surface of the particles even after hardening. In addition to influencing drug diffusion process, the attachment of biotin and the mole percentage of PVA also affect drug release in the burst phase, in which proteins are desorbed from the outer surface of microparticles. Table 3 shows that the attachment of biotin reduces the value of *φ*_d_^burst^, the mass fraction of drug that is desorbed during the burst stage. The authors believe this is due to the fact that the attachment of biotin reduces the amount of BSA trapped on the surface during formation due to steric hindrance. Since the attachment of biotin cannot change the desorption pattern of proteins from the outer surface, it does not affect drug desorption rate constant (*k*_*d*_) and drug induction time (*t*_*d*_). It can also be seen from Table 3 that the mole percentage of PVA has a minor effect on protein desorption during the burst phase. This result, along with the result that the increasing mol% of PVA did not have a drastic effect on the drug loading and/or particle morphology, indicates that the higher mole percentage of PVA is not providing a more stable emulsion during the solvent hardening stage of microparticle synthesis. This also indicates that the increase in PVA mole percentage does not improve the emulsion process, since it cannot guarantee that more of the drug will remain within the microparticle during encapsulation.

**Table 3:** **Parameter estimation results**

Type (mol% PVA)		${\bar{D}}_{d}^{*}$(${\mathrm{cm}}^{2}s^{-1}$)	***φ***_d_^burst^	***K***_d_(day^-1^)	***t***_d_(day)
88	PLGA BSa	1.5 × 10^-13^	0.03	7.60	0.25
	Biotin BSA	9.87 × 10^-14^	0.16	8.10	0.25
98	PLGA BSa	1.38 × 10^-13^	0.28	8.63	0.25
	Biotin BSA	8.37 × 10^-14^	0.22	8.50	0.25

Values of the mass fraction of drug involved in the burst phase ($\varphi_{d}^{\text{burst}}$), drug desorption rate constant (*k*_d_), effective drug diffusivity ($\bar{D_{d}^{*}}$), and drug induction time (*t*_d_) are determined from the drug release profiles.

## Conclusions

Polymeric microparticles created through a water-in-oil-in-water double emulsion effectively demonstrated a controlled release of a model drug. The presence of biotin on the outside of the polymeric microparticles was confirmed using fluorescent imaging. The microparticle synthesis was optimized through the use of PVA consisting of various mole percentages. The effect of the different PVA surfactant on microparticle synthesis determined that 88 and 98 mol% PVA created similar particles that only differed in size and slightly (approximately 10%) in encapsulation. It was determined that increasing the mole percentage of PVA created a more stable emulsion during the hardening phase, allowing for higher encapsulation efficiencies of the model drug (BSA). The release studies found that the attachment of biotin to the PLGA microparticle had only a minor effect on the release trend during the 28-day period. The microparticles still exhibited a controlled release over the 28 days with minimal burst and, therefore, are still believed to be effective as carriers for therapeutic drugs. A release kinetics model was used to further quantify the effective drug diffusivity and drug desorption rate, revealing that the attachment of biotin to microparticles slowed down both drug desorption and drug diffusion processes, while the mole percentage of PVA only has a minor effect on drug release rate.

The presence of the biotin on the microparticle, overall, did not have a negative effect on the microparticles ability to entrap and control the release of the model drug. This indicates that the microparticles can be further investigated for their ability to target using a moiety specific to breast cancer (or other types). This moiety will be attached to avidin and combined with the microparticle using the biotin exposed on the microparticle’s surface. Until that specific moiety is identified, the effect of the avidin-targeting moiety on the microparticle cannot be evaluated. The authors believe that this robust linkage system will be valuable compared to other targeting strategies, since the simple chemistry will allow for linkage of a variety of different moieties. Therefore, the same technology as well as the proposed integrated experimental and modeling approach can be used to target either multiple types of cancer cells, or to include multiple targeting antigens for the same cell on one microparticle.

## Authors’ original submitted files for images

Below are the links to the authors’ original submitted files for images. Authors’ original file for figure 1 Authors’ original file for figure 2 Authors’ original file for figure 3 Authors’ original file for figure 4 Authors’ original file for figure 5
