# Supplementary figures and images for: An integrated experimental and modeling approach to propose biotinylated PLGA microparticles as versatile targeting vehicles for drug delivery
Source: Prog Biomater. 2013 Feb 13;2:3. doi: 10.1186/2194-0517-2-3 (PMC5151110; doi:10.1186/2194-0517-2-3)

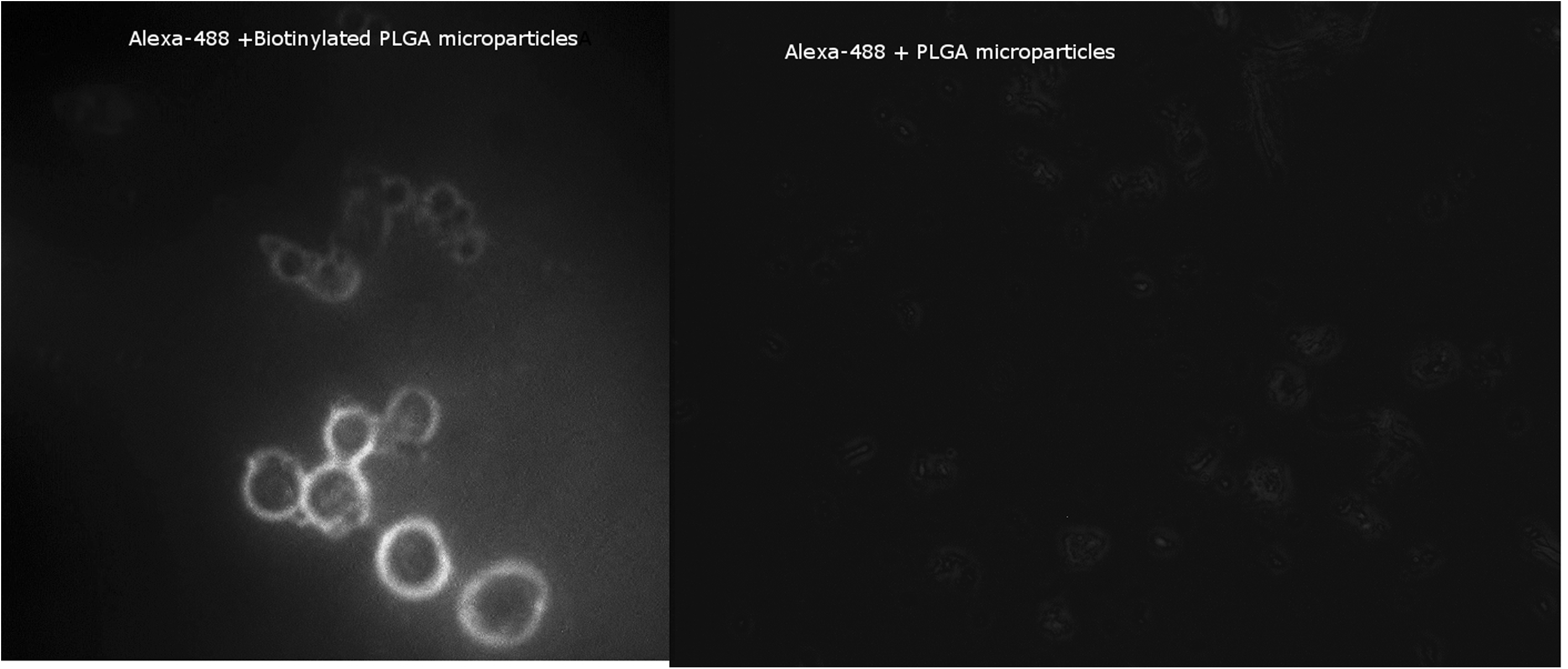

Supplement: Supplementary file 1 — Authors’ original file for figure 1 [file 40204_2012_9_MOESM1_ESM.tiff]

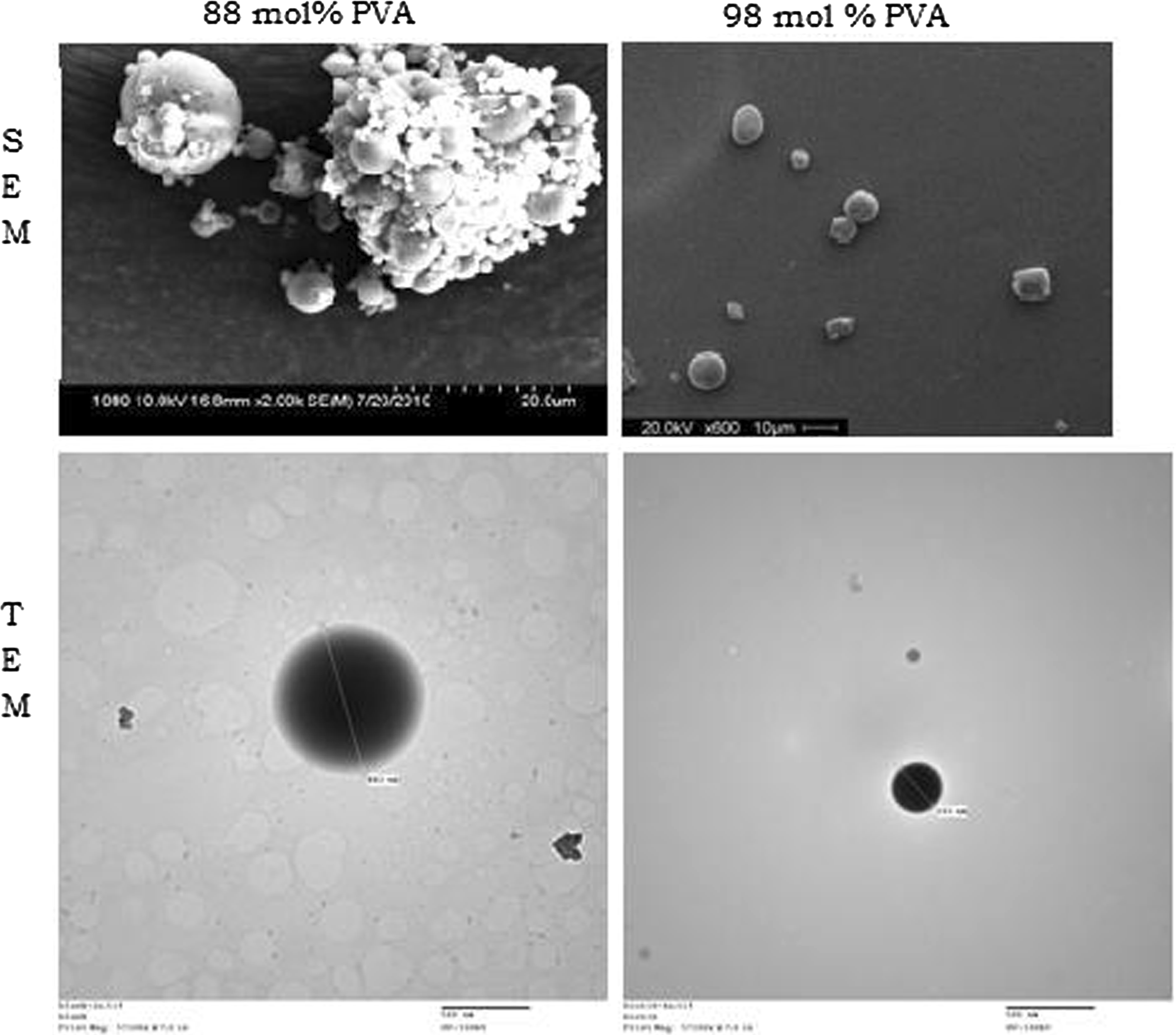

Supplement: Supplementary file 2 — Authors’ original file for figure 2 [file 40204_2012_9_MOESM2_ESM.tiff]

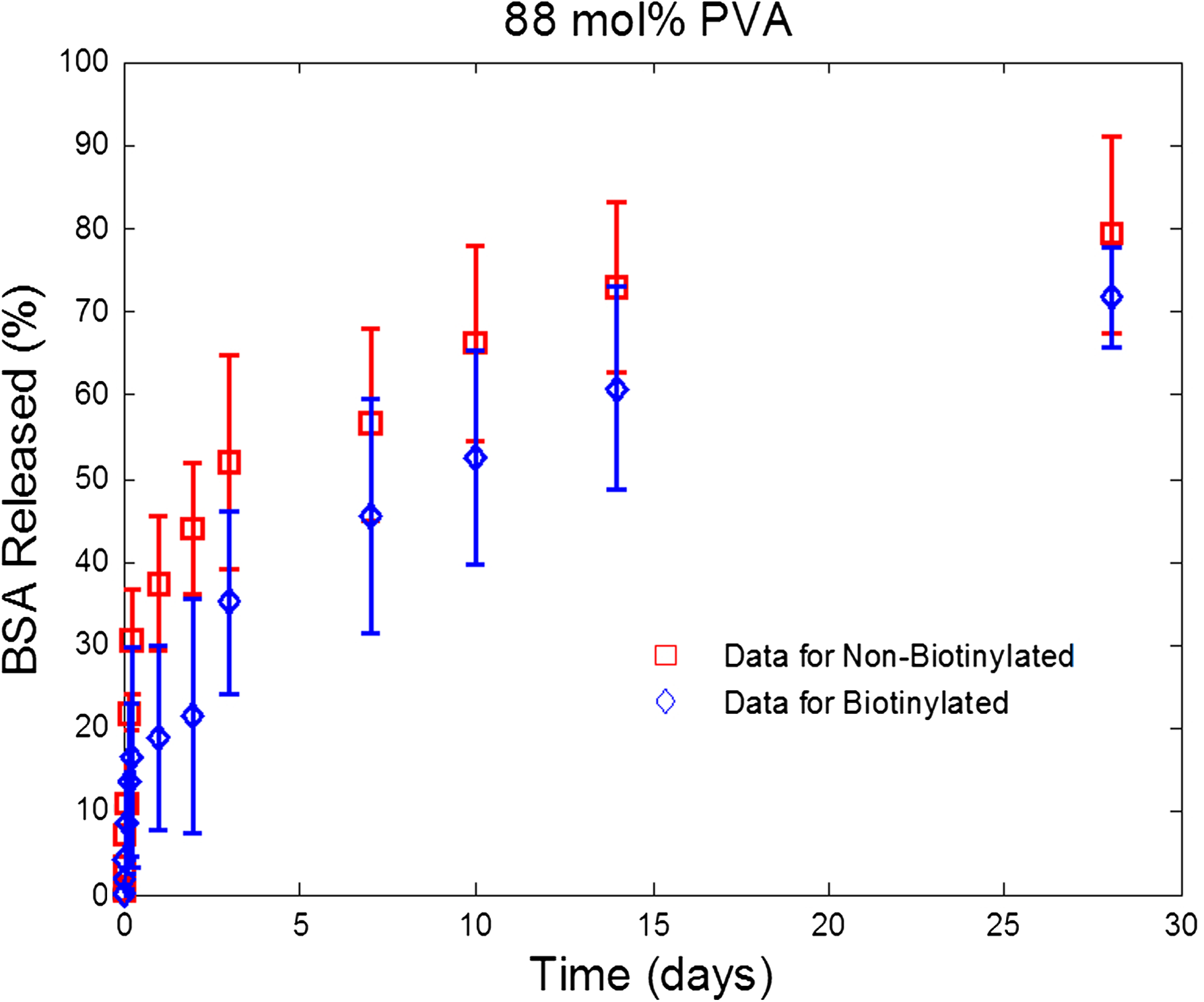

Supplement: Supplementary file 3 — Authors’ original file for figure 3 [file 40204_2012_9_MOESM3_ESM.tiff]

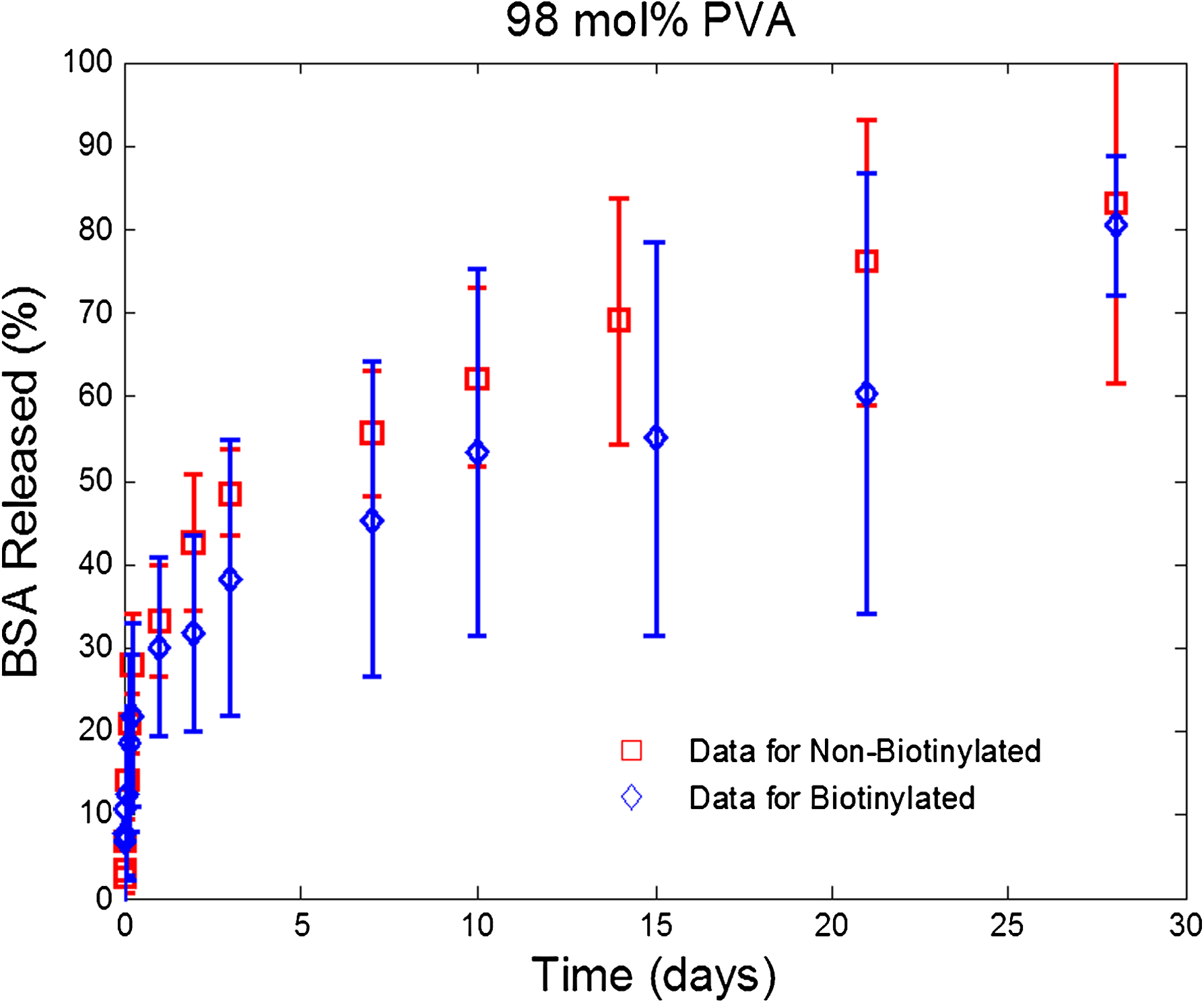

Supplement: Supplementary file 4 — Authors’ original file for figure 4 [file 40204_2012_9_MOESM4_ESM.tiff]

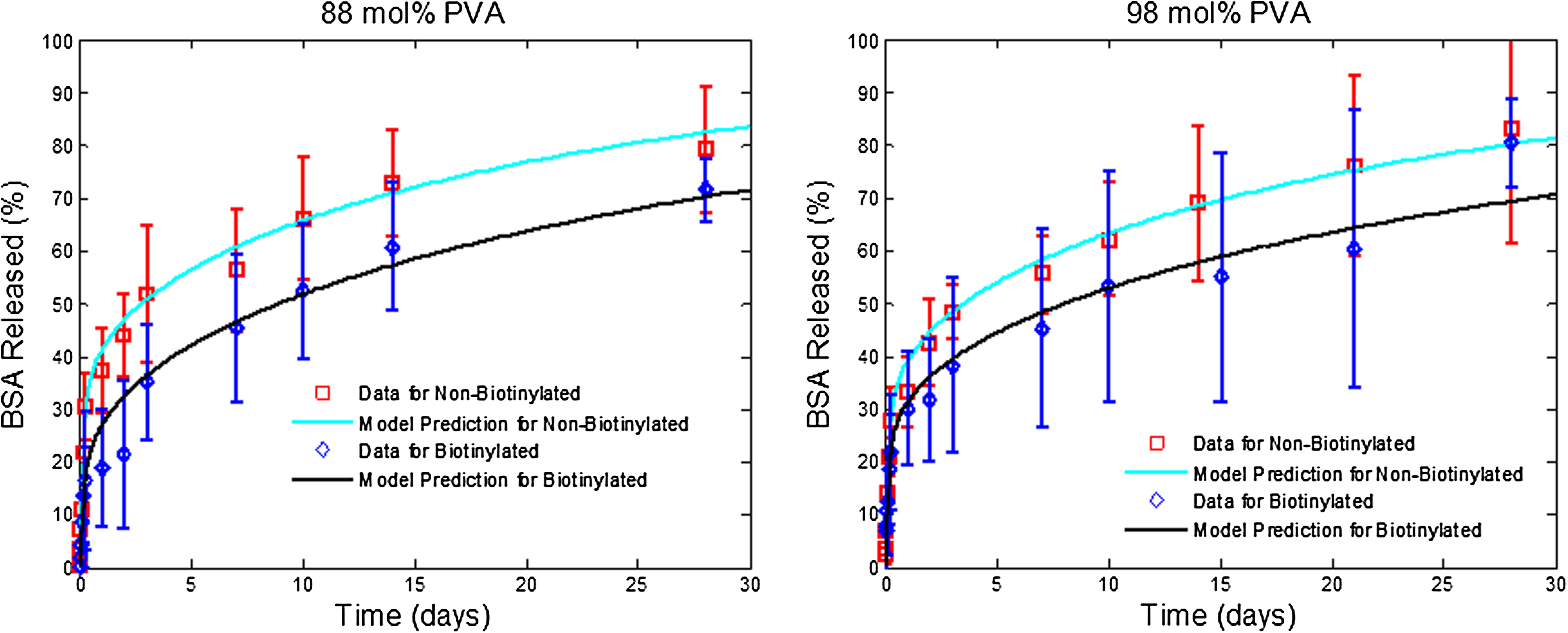

Supplement: Supplementary file 5 — Authors’ original file for figure 5 [file 40204_2012_9_MOESM5_ESM.tiff]
